# A multi-centre prospective development study evaluating focal therapy using high intensity focused ultrasound for localised prostate cancer: The INDEX study^☆^^☆☆^

**DOI:** 10.1016/j.cct.2013.06.005

**Published:** 2013-09

**Authors:** L. Dickinson, H.U. Ahmed, A.P. Kirkham, C. Allen, A. Freeman, J. Barber, R.G. Hindley, T. Leslie, C. Ogden, R. Persad, M.H. Winkler, M. Emberton, on behalf of the INDEX Study Group

**Affiliations:** a Division of Surgery and Interventional Sciences, University College London, UK; b Department of Urology, University College London Hospitals NHS Foundation Trust, UK; c Department of Radiology, University College London Hospitals NHS Foundation Trust, UK; d Department of Histopathology, University College London Hospitals NHS Foundation Trust, UK; e Department of Statistical Science, University College London, UK; f Department of Urology, Basingstoke and North Hampshire NHS Foundation Trust, UK; g Nuffield Department of Surgical Sciences, Oxford University Hospitals, UK; h NIHR Biomedical Research Centre, Churchill Hospital, Oxford, UK; i Department of Urology, Royal Marsden NHS Foundation Trust, London, UK; j Department of Urology, University Hospitals Bristol NHS Foundation Trust, UK; k Department of Urology, Imperial College Healthcare NHS Trust, London, UK

**Keywords:** Focal therapy, High-intensity focused ultrasound, Multi-centre, Prospective study, IDEAL guidelines

## Abstract

**Introduction:**

Focal therapy offers the possibility of cancer control, without the side effect profile of radical therapies. Early single centre prospective development studies using high intensity focused ultrasound (HIFU) have demonstrated encouraging genitourinary functional preservation and short-term cancer control. Large multi-centre trials are required to evaluate medium-term cancer control and reproduce functional recovery. We describe the study design of an investigator-led UK multi-centre, single arm trial using HIFU to deliver focal therapy for men with localised prostate cancer.

**Methods:**

One-hundred and forty men with histologically proven localised low or intermediate risk prostate cancer (PSA < 15, Gleason ≤ 7, ≤ T2cN0M0) will undergo precise characterisation of the prostate using a combination of multi-parametric (mp)MRI and transperineal template prostate mapping (TPM) biopsies. Unilateral dominant tumours, the so-called index lesion, will be eligible for treatment provided the contra-lateral side is free of ‘clinically significant’ disease (as defined by Gleason ≥ 7 or maximum cancer core length ≥ 4 mm). Patients will receive focal therapy using HIFU (Sonablate 500®). Treatment effect will be assessed by targeted biopsies of the treated area and TPM biopsies at 36-months.

**Results:**

Primary outcome is the absence of clinically significant disease based on 36-month post-treatment TPM biopsies. Secondary outcomes address a) genitourinary function using validated patient questionnaires (IPSS, IPSS-QoL, IIEF-15, EPIC-Urinary, EPIC-Bowel, FACT-P, EQ-5D), b) the predictive validity of imaging, and c) risk factors for treatment failure.

**Conclusions:**

INDEX will be the first multi-centre, medium term follow-up trial to evaluate the outcomes of a tissue preserving strategy for men with localised prostate cancer using the TPM-ablate-TPM strategy.

## 1 Introduction

Focal or tissue-preserving therapy is a strategy that offers men the potential for treating their localised prostate cancer with a lower side-effect profile [1–4]. At present, men can expect 30–90% erectile dysfunction, 5–20% incontinence and 5–20% rectal toxicity from radical prostatectomy or radiotherapy [5–7]. These over-treatment harms may not be acceptable in light of the small treatment benefit that can be derived [8–10]. Early results from a number of small single centre studies evaluating focal therapy have reported urinary incontinence in about 1% and erectile dysfunction in 5–10% of men with good baseline function [2,3,11].

However, these results have limited external validity since they may be the product of careful patient selection, expert treatment in specialist centres, and surrogate outcomes derived over a short time-frame [12]. The next phase of development therefore requires evaluation of this complex intervention within a multi-centre setting, with longer follow-up, and with primary outcomes based on disease control.

Although this is a laudable aim, there are difficulties in designing a trial to evaluate outcomes on disease control. Firstly, prostate cancer has a prolonged natural history. If overall and disease-specific mortality were used as primary outcomes, for instance, this would require hundreds of patients recruited over many years and followed for at least 10–15 years to obtain any degree of precision. Secondly, as multi-focal disease is present in most cases of prostate cancer, focal therapy inherently involves ablation of only the dominant area, leaving behind tissue that is likely to harbour prostate cancer lesions. Benign tissue may also be predisposed to develop lesions *de novo* through a field effect. Measuring rates of progression of untreated tissue requires novel approaches. Thirdly, there exists no consensus on the optimal medium term endpoints in tissue preserving therapy since those surrogate measures used in radical whole-gland therapies, which are primarily serum prostate specific antigen (PSA) based, cannot be readily translated to a treatment paradigm in which 50% or more of the prostate tissue is still present [13,14]. Indeed, the FDA in the US has failed to devise a regulatory pathway for this increasingly adopted form of treatment.

The design of INDEX was informed by the reports of earlier registered studies (NCT00561314, NCT00561262) that evaluated different approaches to focal therapy, and from a number of pivotal consensus meetings and processes [15–17]. This report constitutes the next phase of the IDEAL development pathway of a surgical intervention [18] and the MRC (UK) guidelines [19] on evaluating a complex intervention.

## 2 INDEX study protocol

### 2.1 Study management

INDEX is a prospective, multi-centre, single-arm, therapeutic, investigator-led study, conforming to Stage 2B of the IDEAL clinical trial guidelines for evaluation of a surgical intervention [18]. It is sponsored by University College London, with commercial support from SonaCare Medical LLC (Charlotte, North Carolina, USA), distributors of the Sonablate 500® device, for infra-structural study costs (such as study personnel, transport of device, trial meetings) through an unrestricted grant made to UCL. The trial protocol was designed by investigators from University College London, with input from external peer reviewers and patient representatives, and conducted according to Good Clinical Practice (GCP) guidelines. Monitoring of subject safety and study compliance is being managed by Data Monitoring and Trial Steering Committees, comprising an impartial (medically qualified) chairperson, the co-chief investigators, study coordinator, principal investigators from each study site, study statistician, and two patient representatives.

### 2.2 Study population

Since June 2011, INDEX has been recruiting men with histologically confirmed, localised low or intermediate risk prostate cancer (PSA < 15, Gleason ≤ 7, ≤ T2cN0M0) on transrectal ultrasound guided (TRUS) biopsies or template prostate mapping (TPM) biopsies (using a 5 mm sampling frame), who have not previously undergone treatment. The recruiting centres are University College London NHS Foundation Trust (sponsor centre), Hampshire Hospitals NHS Foundation Trust, Imperial College Healthcare NHS Trust, Oxford University Hospitals NHS Trust, Royal Marsden NHS Foundation Trust, and University Hospitals Bristol NHS Foundation Trust.

### 2.3 Eligibility

**Fig. 1:**
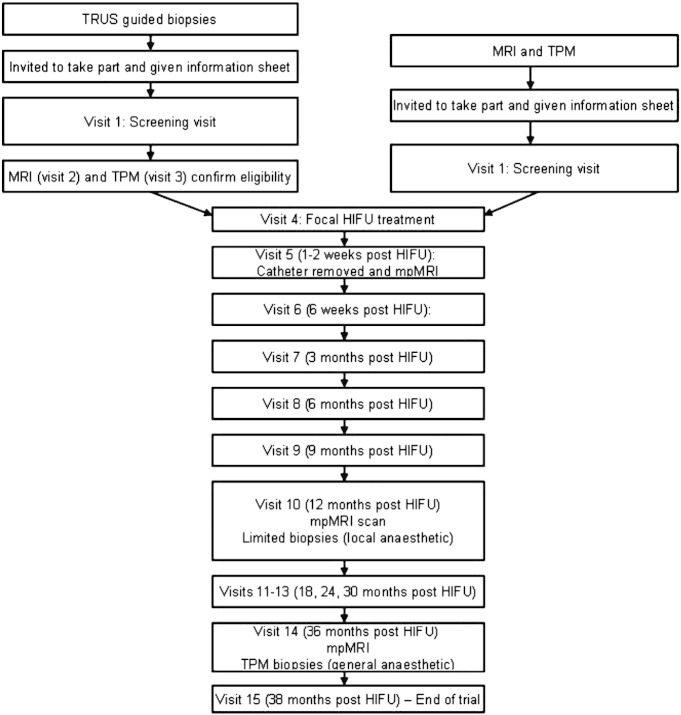
Trial flow.

Men are considered eligible for the trial if they have unilateral or bilateral disease on TRUS biopsy, with a maximum 3 mm Gleason 3 + 3 disease on the non-dominant side, and/or either unilateral disease or bilateral disease on TPM with no more than clinically insignificant disease on the non-dominant side (i.e. outside of the planned treatment area). Men recruited following diagnostic TRUS biopsy undergo mpMRI and TPM prior to focal HIFU treatment, to accurately map and locate disease, and to ensure eligibility. Men can also be recruited and proceed straight to focal HIFU treatment if they have already undergone mpMRI and TPM that conform to the INDEX standards of conduct and reporting (Fig. 1). Full eligibility criteria are detailed in Appendix A.

### 2.4 Study design

#### 2.4.1 Trial entry

**Fig. 2:**
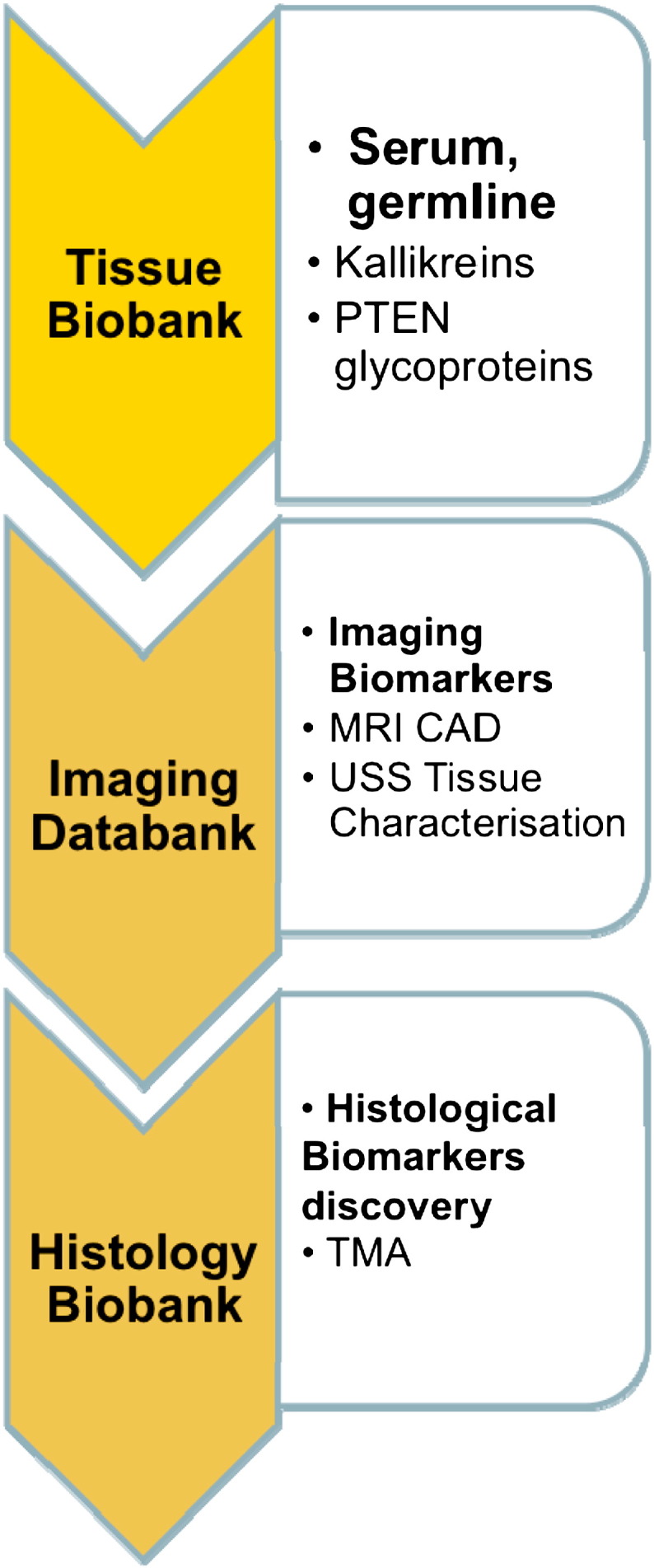
Imaging and pathological databanks.

Eligible men are offered a patient information sheet, and are invited to attend a screening visit. Those meeting inclusion and exclusion criteria are fully counselled to their treatment options before the screening visit and informed consent. This is part of the UK multidisciplinary approach to cancer management. Validated patient questionnaires (International Prostate Symptom Score (IPSS), International Prostate Symptom Score-Quality of Life (IPSS QoL), International Index of Erectile Function-15 (IIEF-15), UCLA Expanded Prostate Cancer Index Composite (EPIC) urinary and bowel domains, EQ-5D Quality of Life, Functional Assessment of Cancer Therapy (FACT)-Prostate, and Memorial Anxiety Scale for Prostate Cancer) are completed at baseline. Serum blood tests including PSA, renal function, full blood count, and any other tests required to assess fitness for general anaesthetic are performed. Patients are also asked to consent (optional) to additional urine and blood samples for the purpose of biobanking in order to develop and validate novel biomarkers. Those consenting are asked to provide samples at baseline, 12 months and 36 months (Fig. 2). The translational objectives of these research samples are subject to planned academic collaborations, study protocols, and ethics approvals.

#### 2.4.2 Disease localisation

There is lack of consensus on the optimal strategy used to localise individual lesions of cancer. Both mpMRI and TPM have been proposed individually, and in combination. State-of-the-art mpMRI has a very high negative predictive value (in the order of 95%) for clinically significant disease [20–22] and therefore could be used to determine which areas of prostate do not undergo treatment. However, there is an additional requirement for histological verification of both the dominant lesion (since the positive predictive value for mpMRI is at present not high) and absence of clinically significant disease (as defined by Gleason ≥ 7 or maximum cancer core length ≥ 4 mm) in the untreated area. As a result, INDEX will use both tests in combination.

##### 2.4.2.1 Imaging

Staging investigations follow local cancer network guidelines. Multi-parametric MRI is performed prior to TPM biopsies, and at least 6 weeks after any previous diagnostic biopsy, in order to limit biopsy artefact that may affect image interpretation. Pre-operative, and all post-HIFU, imaging is performed using either a 1.5 Tesla or 3 Tesla MR scanner, and a pelvic phased array receiver, with a pelvic coil. A full protocol of T1 and T2 weighted turbo-spin echo images and a dynamic post gadolinium volume acquisition is used for both pre-operative diagnostic and planning scans and post-operative assessment of focal treatment effect. The protocol is detailed in Appendix B.1. A 5-point Likert-type scoring system is used to report the probability of malignancy from the images (Appendix B.2), as described from a European consensus meeting on prostate mpMRI [23,24]. The results are conveyed in diagrammatic, number and written form using a standardised proforma. The prostate is divided into 27 Regions of Interest for scoring. An example reporting form is provided in Appendix B.3.

##### 2.4.2.2 TPM biopsies

**Fig. 3:**
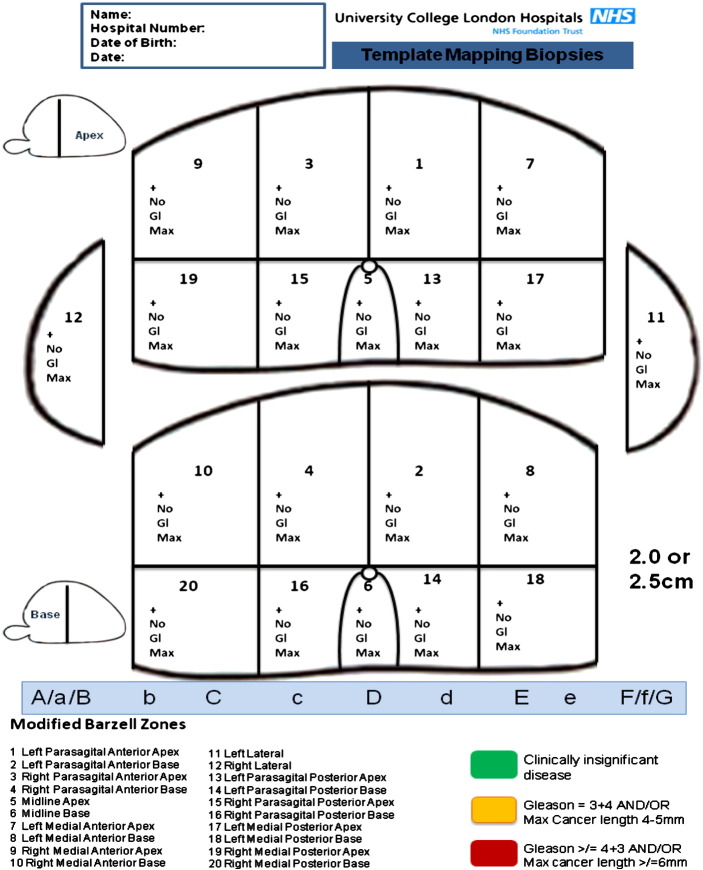
Transperineal template mapping biopsy reporting protocol.

The process by which the specified distribution of cancer is verified is primarily on transperineal template 5 mm spaced prostate mapping biopsies. Biopsies are taken every 5 mm from the prostate using a brachytherapy grid placed over the perineal skin, with the patient in the lithotomy position. The number of biopsies is otherwise not defined, and is dependent on prostate size. 3-Dimensional data on the location and specific grade for each focus of cancer is produced within pictorial (Fig. 3) and written reports and focal ablation planning based on this information. TPM biopsies have a high accuracy for clinically significant lesions with 95% sensitivity and 95% negative predictive value for those lesions of 0.5 cm^3^ or greater in volume [25,26].

#### 2.4.3 Focal therapy intervention

##### 2.4.3.1 Ablative modality

HIFU works by focusing and depositing a large pulse of high-energy ultrasonic waves on a single area, thereby increasing the temperature to a point whereby it causes coagulative necrosis. Focused ultrasound waves are emitted from a transducer and are absorbed in the target area of approximately 3 × 3 × 10 mm of tissue. The result is a targeted thermal effect with minimal, or no, damage to the tissue in the path of the ultrasound beam [27]. Two commercially available devices exist for HIFU therapy: Ablatherm (Edap Technomed, Vaulx-en-Velin, France) and Sonablate 500® (Focus Surgery, Indianapolis, IN, USA). This study uses the Sonablate 500® device, which has a therapy-imaging transducer with different focal lengths, and user-dependent delivery of treatment according to live ultrasound images, allowing precise control of energy delivery by each pulse. Our reason for choosing this device is due to prior expertise developed in its use for whole-gland ablation of the prostate [28].

##### 2.4.3.2 Treatment protocol

HIFU treatment is performed as a day-case procedure, unless travel distance or co-morbidities indicate an overnight stay, under general or regional anaesthesia. A transrectal resection of the prostate (TURP) is not required prior to treatment, although permitted if performed at least 6-months prior to study recruitment. A suprapubic catheter is inserted under cystoscopic guidance prior to treatment, or a urethral catheter at the end of treatment if supra-pubic insertion is contra-indicated. A suprapubic catheter has previously been shown by our group at UCLH to reduce urethral stricture rates [28]. Patients are discharged with the suprapubic catheter on free drainage for 24–48 h, with a planned trial without catheter between 5 and 14 days post-operatively. They are prescribed simple analgesia (Diclofenac or Co-dydramol), laxatives, and a short course of quinolone antibiotics (ciprofloxacin) at the clinician's discretion.

**Fig. 4:**
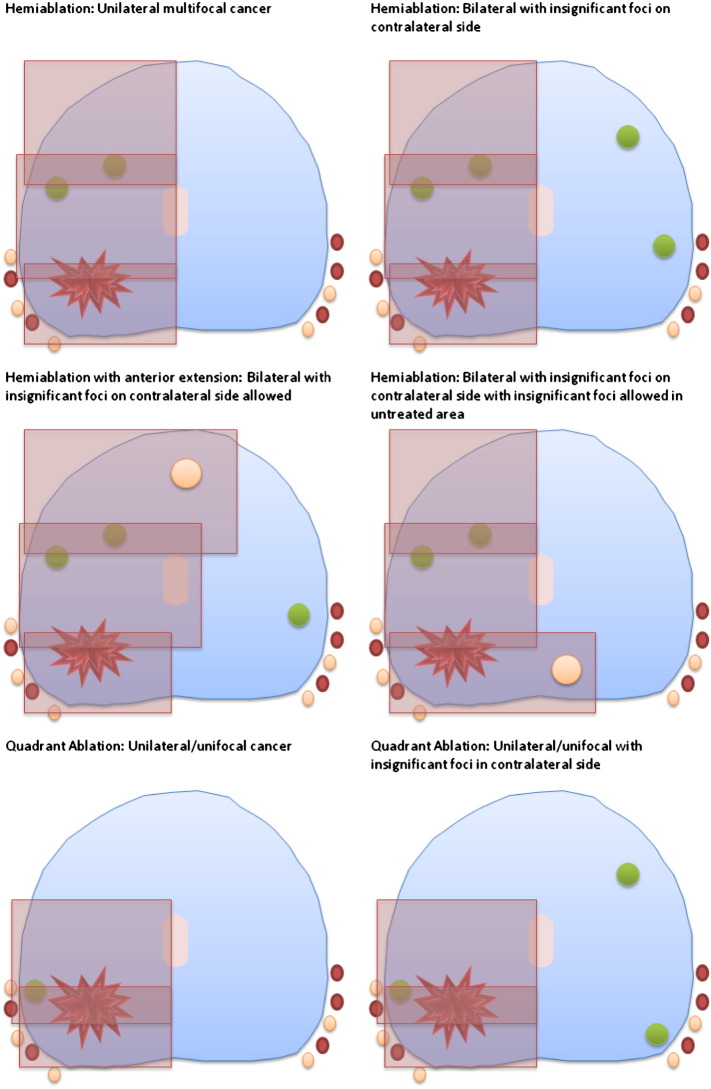
Treatment protocol.

The treatment is delivered to the hemi-gland (left or right) in which the index lesion(s) has been identified by a combination of mpMRI and TPM biopsy (Fig. 4). Treatment can extend posteriorly or anteriorly over the midline, if required, up to a maximum of 60% tissue ablation, and is standardised to a hemi- or extended hemi-ablation in centres with surgeons that have performed fewer than 20 cases independently, in order to standardise delivery during the learning curve stage. Experienced centres may ablate a quadrant. Treatment is planned to reach the urethra and may cross the midline by up to 5–10 mm if the disease is close to, or crosses, the midline. At least one neurovascular bundle is avoided by ensuring a minimum distance of ablation zone of 10 mm. One redo-HIFU to the treated side is permissible, as per current protocols and standard practice for HIFU, if either 12-month targeted biopsies of the treated side or ‘for-cause’ biopsies are positive, up until trial exit at 36-months.

### 2.5 Follow-up visits

Trial clinic visits (telephone or clinic consultation) occur at 6 weeks, 3, 6, 9, 12, 18, 24, 30 and 36 months. At each visit data is collected on adverse events, patient questionnaires, and serum PSA levels. An early contrast-enhanced MRI is performed at 1–3 weeks post focal HIFU to verify that the treatment has been delivered appropriately according to plan as well as to determine whether adequate energy has been delivered. It is carried out in the first 5 patients per centre (at least) as a quality control measure as it shows areas of perfusion deficits that usually correlate well with the quality of treatment delivery [29].

Men receive further mpMRIs (as per pre-HIFU protocol) at 12 and 36-months. Targeted biopsies are taken at 12-months under local anaesthetic, using the transrectal or transperineal route, as per clinician's discretion. These are of the treated area only in order to assess treatment success, with a minimum of 1 biopsy per 1–2 ml of prostate volume. Biopsy of the untreated area is only carried out if a new suspicious lesion is detected on the 12-month mpMRI, which was not present in that area on the pre-treatment mpMRI. This limited biopsy protocol is carried out in order to minimise burden on the patient, especially as a full mapping had been conducted only 12 months prior to this, and it would be rare for any untreated tissue to progress within such a timeframe. At 36-months, a further TPM is carried out of all residual tissue, and reported in the same format as the pre-treatment template biopsies in order to measure progression, if any. Areas that are absent within the 20-zone protocol are omitted.

#### 2.5.1 For-cause tests

Clinicians can biopsy the prostate between primary focal HIFU and 36 months if there is a clinically significant rise in PSA (‘for cause’ biopsy) following consensus approval by a Study Investigators Group. In the event of apparent significant under-treatment on early mpMRI, an additional ‘for-cause’ mpMRI may be performed a minimum of 6-months following HIFU, at the discretion of the Study Investigators Group. This is the first time-point at which prostatic inflammation is expected to have diminished sufficiently to detect any foci of residual disease, potentially warranting ‘for-cause’ biopsy. Other ‘for cause’ additional tests such as ultrasound, mpMRI, CT scan, bone scan or PET scan are permissible as per local centre practice.

### 2.6 Objectives

There are two co-primary objectives of INDEX for cancer control on the 36-month TPM. The first is to determine the proportion of men who are free of *any* prostate cancer in the treated area and are free of *clinically significant* prostate cancer in the untreated area 36 months after focal therapy using HIFU. The second is to determine the proportion of men who are free of *clinically significant* prostate cancer in the treated area and are free of *clinically significant* prostate cancer in the untreated area 36 months after focal therapy using HIFU.

Secondary objectives include an assessment of interim cancer control at 12 months as assessed on targeted biopsy of the treated area, short to medium-term functional (sexual, urinary, bowel) and quality of life outcomes, the rate of secondary prostate cancer intervention (prostatectomy, radiotherapy, androgen ablation, whole-gland HIFU or cryosurgery), and an assessment of biochemical (PSA) kinetics, following focal HIFU. Risk factors for failure to achieve the co-primary objectives will be analysed. Finally, an assessment of the clinical validity (sensitivity, specificity, negative and positive predictive values, inter-observer variability) will be made of the mpMRI imaging technique both to identify the presence of clinically significant prostate cancer on TPM biopsies prior to focal therapy, and the presence of residual/recurrent clinically significant prostate cancer on post-HIFU (12-month and 36-month) biopsies.

At the lead centre only (University College London Hospital), an additional nested pilot study has been performed in the first 26 patients treated with an MR-visible lesion, on the safety and feasibility of a novel MR-TRUS registration system for planning and conducting focal treatment of prostate cancer [30]. The MR-US registration system has previously been described by Hu et al. [31,32]. The secondary objectives of this pilot study were to determine the number of patients in whom the planned treatment volume was increased as a result of MR-US image registration, the volume change between initial and registration-informed treatment plans, the time required to plan the treatment manually versus registration-based planning alone or a combination of the two methods, and the volume overlap between target volumes, as defined by the HIFU treatment plan, and the regions of necrosis visible in post-operative MR images. Full results of this pilot study have been recently published elsewhere [30].

Pending financial resources, we aim to determine the costs of focal treatment, with modelling of potential cost effectiveness using existing datasets of cancer control and functional outcomes at 36 months achieved by radical whole-gland therapies and active surveillance.

### 2.7 Training/quality control protocol

The success or otherwise of a new intervention is heavily dependent on training and quality control of new users. This needs to be both comprehensive and flexible, to fit in with clinical practice. With these factors in mind a pragmatic, but nonetheless robust, clinical training programme has been drawn up for the purpose of delivering the interventions within this trial. Only clinicians attending the training sessions (or another equivalent training programme) are approved as reporters or surgeons within the study.

#### 2.7.1 Multi-parametric MRI

A nominated consultant radiologist attended the lead centre for a training day on the conduct and reporting of pre- and post-treatment (early and late) mpMRI prior to the study commencing. The mpMRI from the first 5 patients are double reported by a radiologist expert in prostate MRI as a quality control measure. Discrepancies are dealt with by an arbitrating third radiologist, expert in prostate MRI.

#### 2.7.2 TPM biopsies

All clinicians carrying out TPM biopsies are required to carry out TPM biopsies to protocol standard. Each clinician is required to observe a minimum of two TPM biopsies at an expert centre. Each clinician will then be proctored for the first two cases by an approved expert proctor, with the period of proctoring extended at the discretion of the proctor.

#### 2.7.3 TRUS guided biopsy

There is no formalised specific training programme for clinicians carrying out TRUS biopsies but all clinicians are required to conduct the targeted TRUS biopsies after focal therapy as laid down by the trial protocol.

#### 2.7.4 Pathology

A specialist pathology meeting was held prior to trial commencement, for nominated prostate histopathologists with expertise in uropathology, where the requirements for standardised pathology reporting were discussed. All study pathologists are required to report the biopsies as laid down by the trial protocol.

#### 2.7.5 Focal HIFU

Clinicians undergo training and proctoring to ensure treatment is delivered to a standard laid down by the lead centre. Clinicians with or without previous HIFU experience are required to visit an expert training centre on at least two occasions and observe at least three cases of focal HIFU. Clinicians with previous HIFU experience are required to undergo proctoring for at least their first 5 cases on any number of visits, with extension at the discretion of the proctor. Clinicians with no previous HIFU experience are required to undergo proctoring for their first ten cases on any number of visits, with extension at the discretion of the proctor. Clinicians are signed off for non-proctored cases once the first 10 or 20 cases for that clinician has undergone review, including against post-treatment early contrast MRI. Only approved clinicians deliver the treatment within this trial. Only one clinician per site is proctored until competent to perform focal HIFU independently. Each trial centre is required to treat at least 5 patients within the trial period to ensure that the required HIFU treatment skills are maintained. Any re-do focal HIFU treatments performed within the trial period will be proctored with an expert proctor and/or HIFU technician present.

### 2.8 Statistical analysis

#### 2.8.1 Sample size calculation

The primary objective is to estimate the proportion of men who are free of *any* prostate cancer in the treated area and are free of *clinically significant* prostate cancer in the untreated area 36 months after focal therapy using HIFU. The second is to determine the proportion of men who are free of *clinically significant* prostate cancer in the treated area and are free of *clinically significant* prostate cancer in the untreated area 36 months after focal therapy using HIFU. Evidence from a small single centre trial at UCLH demonstrated that event rates could be as high as 100% absence of clinically significant cancer and 90% absence of any cancer in the treated areas at 6 months following focal therapy [2]. Clinical knowledge indicates that these rates are likely to be lower in a multi-centre trial with further follow up. In calculating sample size for the current study we therefore assumed 90% of patients will have no evidence of clinically significant cancer at 36 months and 80% will have no evidence of any cancer at 36 months in the treated area. Using a precision-based calculation for 95% confidence intervals we calculated that at least a sample size of 140 patients would be needed to estimate these proportions to within 7% (including an inflation for 10% dropout) [33].

#### 2.8.2 Primary outcomes

The proportion of patients with evidence of clinically significant prostate cancer at 36 months and those with no evidence of cancer at 36 months (in the treated areas as per the outcome definitions above) will be estimated along with associated 95% confidence intervals.

#### 2.8.3 Secondary outcomes

Secondary outcomes will be reported as estimates with 95% confidence intervals calculated using standard statistical methods as appropriate for the type of outcome. For patient reported outcomes with available baseline measurements, comparison will be made with baseline values using paired analyses. Logistic regression will be used to investigate associations with potential risk factors for histological failure, considering PSA, Gleason score, cancer core length involvement (mm and %), number and % of positive biopsies for any cancer on TPM and TRUS, stage and D'Amico risk group (low, intermediate, high). Sensitivity and specificity (with 95% confidence intervals) will be estimated in considering the use of standard PSA kinetics and thresholds for identifying clinically significant cancer. Modelling methods for serial measurements will be used to consider patterns of PSA change associated with subsequent positive biopsy.

## 3 Discussion

### 3.1 Summary of protocol

The INDEX trial will be the first prospective study testing focal therapy with HIFU within a multi-centre setting, with medium-term quality of life and histological outcomes. The safety and tolerability of focal HIFU within single centre studies are already known [2,3]. These demonstrate a very low event rate for both erectile dysfunction and incontinence, and encouraging cancer control, over a 12-month follow-up period. It follows that INDEX should be the next step within a phased development and evaluation programme.

We have used a pragmatic trial design, to include men with a range of baseline functional status. Furthermore, we have included men with unilateral/unifocal disease and those with multi-focal disease with treatment targeted to the index lesion. There is a new body of evidence emerging demonstrating that the index lesion usually harbours the highest Gleason pattern, and is responsible for disease progression [34–39]. There have been recent calls to re-assign low volume low grade lesions as something other than cancer in order to reflect their indolent nature [40–42]. INDEX follows a ‘template–focal treatment–template’ protocol, ensuring highly accurate histological planning and follow-up. This is the first prospective study, to the best of our knowledge, which incorporates 5 mm transperineal template mapping biopsy at study entry and study exit, providing a unique histopathological dataset and the means for interrogating the natural behaviour of untreated benign tissue and clinically insignificant disease [43].

INDEX may also confirm that focal therapy can lead to low rates of genitourinary and rectal toxicity and minimal impact on quality of life within a large and more representative cohort of patients than in previously described studies. Further, an important step in the dissemination of a new technique is the transfer of skills within a safe and controlled environment. INDEX aims to demonstrate that the diagnostic and therapeutic skills acquired by one research centre are transferable to others. Finally, we aim to estimate costs of care and to model potential cost-effectiveness in comparison to alternative ‘standard’ therapies. If this single arm intervention study demonstrates acceptable outcomes supporting the findings of the early short-term studies it could lead onto a randomised controlled trial, prior to more widespread use of this technology.

### 3.2 Limitations

The first limitation relates to study design. Verification of a new therapy as favourable, or equivalent, to ‘standard’ care is ideally sought through comparison with another matched control group. Randomised controlled trials (RCTs) offer the best method for minimising systematic bias and revealing the true effect of an intervention or drug. However, RCTs involving treatments of localised prostate cancer have had a historically poor patient uptake, as the reference ‘gold’ standard of care is not known. In addition, RCTs are expensive to run and involve huge infra-structural support. A number of trials have been forced to close due to lack of recruitment [44–55]. A randomised trial may be feasible if a pragmatic design is adopted, but prior to acceptance of such a design, the number of centres with expertise in this complex intervention (mpMRI, TPM, focal HIFU) needs to be increased. Observational studies, such as INDEX, are a commonly used alternative to ascertain the effectiveness of a treatment. They are used to observe a treatment effect in a selected group of patients who are presumed to derive benefit from the treatment given. Although methodologically not as robust, and therefore prone to bias, they have some benefits over RCTs. The principal ones are those of enhanced external validity (many patients do not wish to be randomised and therefore refuse participation in RCTs), and more rapid accrual compared to a randomised design. For these reasons, INDEX was designed as a single arm medium term follow-up cohort intervention study, although we acknowledge that it should ultimately lead onto an RCT of focal therapy against ‘standard care’.

Another limitation of INDEX relates to the use of surrogate outcome measures. There are currently no validated or agreed outcome measure other than prostate cancer related deaths or rate of metastatic disease that serve as a meaningful clinical outcome measure across the prostate cancer therapies, including focal therapy. However, in a focal therapy trial with a very low expected rate of death and metastatic progression in a sub-set of low-intermediate risk patients, some 10–15 years would have to pass after treatment before sufficient events were accrued in order to gain meaningful results on which to base the outcomes of this therapy. Furthermore, a trial would require 2000–3000 patients. We have therefore had to adopt surrogate secondary outcome measures of outcome. We are exploring four main outcome categories in this study that we believe will provide optimal and appropriate information at this stage of evaluation of an innovative technique, although all aspects carry some limitations. Firstly, treatment related side-effects will be relatively well captured using validated questionnaires. In focal therapy studies reported to date, stability in terms of functional health status is achieved between 3 and 6 months following the intervention. This domain of outcome will therefore be derived relatively early. The second relates to a more global assessment of quality of life. We are using some tools that are generic, and some designed specifically for the evaluation of patients with prostate cancer. The third, and most problematic area, relates to the type and timing of the surrogate cancer related outcomes used (PSA, biopsy, imaging, additional therapy). This problem arises since radical therapies use very different outcome measures based on PSA kinetics with little consensus across different modalities of treatment. No such outcome measures have yet been validated for focal therapies. Indeed, focal therapy is further problematic in this respect as tissue is left untreated, and this tissue will inevitably give rise to PSA increases with time. The fourth relates to the costs of care and incorporates cost-effectiveness, cost utility and cost benefit. Apart from cost minimization exercises nested on the intervention versus known costs of alternative intervention, most economic analyses will require that cancer outcomes are derived as well as functional status and quality of life. We are collaborating with health economic experts and lead clinicians in this field with the aim of exploring this.

### 3.3 Expected time-frame for results

The first patient was recruited to the INDEX study in June 2011 and received focal HIFU treatment in July 2011. All 6 study centres were actively recruiting patients from May 2012. Recruitment rates have been achieved at the expected accrual rate, and the study is expected to continue recruitment until the third quarter of 2013, with the planned study number of 154 men treated expected by the end of 2013.

## 4 Conclusions

INDEX will test focal therapy within a multi-centre setting, using robust quality control processes to ensure interventions are delivered to a uniform standard. The protocol offers an opportunity to evaluate the natural history of untreated low-grade, low-volume prostate cancer lesions and the index lesion hypothesis. Further, there is a unique opportunity to validate novel imaging and tissue biomarkers as predictors of outcome. It is hoped that the outcomes of this study will lead to further evaluation of focal therapy within a randomised comparative setting prior to widespread dissemination of this technique.

## Figures and Tables

**Figure:**
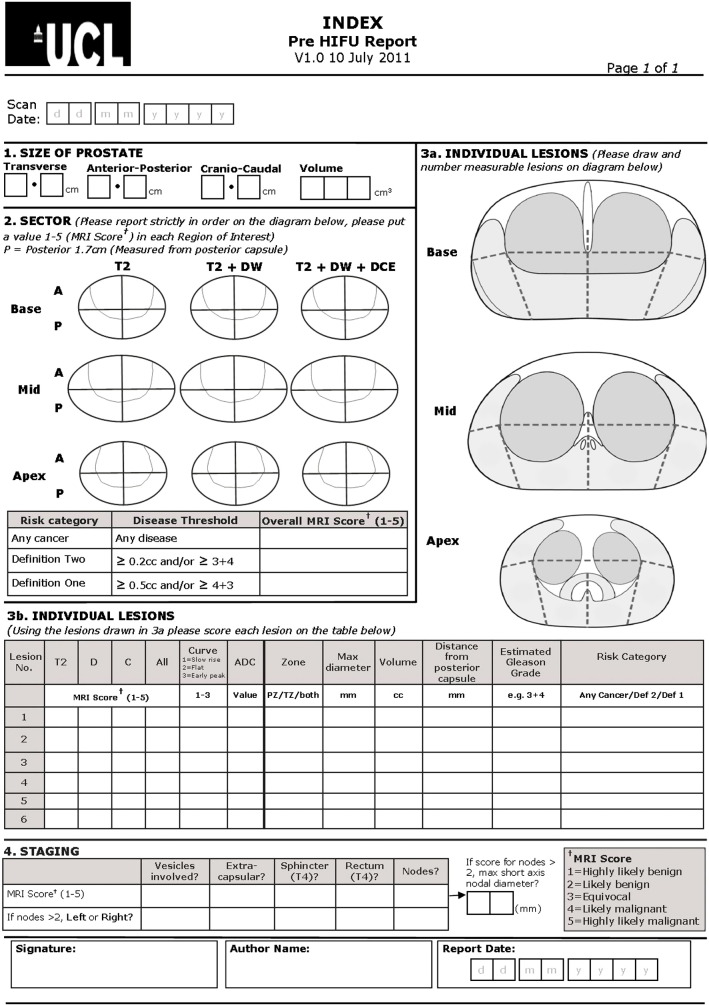


## Appendix A Eligibility criteria

### A.1 Inclusion criteria

The population studied will be those patients who have:

• Histologically proven prostate cancer on trans-rectal or transperineal template prostate biopsies
• TRUS biopsy: up to burden bilateral disease with maximum 3 mm one biopsy on non-dominant side is allowable.
• Template biopsy:
  – unilateral disease any burden
  – bilateral disease
    the presence of clinically significant cancer on only one side (as determined by histological rules described above) Gleason ≤ 7, or
    clinically insignificant disease with a burden of > 50% of biopsy cores taken on that side, or
    bilateral clinically insignificant disease and < 50% of biopsy cores positive on any one side but with dominant disease burden on one side
• Stage T1-T2cN0M0 disease, as determined by local guidelines (radiological T3a permitted)
• Serum PSA ≤ 15
• Life expectancy of ≥ 10 years
• Signed informed consent by patient
• An understanding of the English language sufficient to understand written and verbal information about the trial and consent process.

### A.2 Exclusion criteria

• Men who have had previous radiation therapy
• Men who have had androgen suppression/hormone treatment within the previous 12 months for their prostate cancer
• Men with evidence of metastatic disease or nodal disease outside the prostate on bone scan or cross-sectional imaging
• Men with an inability to tolerate a transrectal ultrasound
• Men with latex allergies as the HIFU probe is covered with a latex condom sheath prior to insertion into the back passage
• Men who have undergone prior significant rectal surgery preventing insertion of trans-rectal HIFU probe (decided on the type of surgery in individual cases)
• Men who have had previous HIFU, cryosurgery, thermal or microwave therapy to the prostate.
• Men who have undergone a transurethral resection of the prostate (TURP) for symptomatic lower urinary tract symptoms within 6 months. These patients may be included within the trial if deferred from consenting and screening until at least 6 months following the TURP.
• Men not fit for major surgery as assessed by a Consultant Anaesthetist
• Men unable to have pelvic MRI scanning (severe claustrophobia, permanent cardiac pacemaker, metallic implant etc. likely to contribute significant artefact to images)
• The presence of metal implants/stents in the urethra
• The presence of prostatic calcification and cysts (on transrectal ultrasound) whose location will interfere with effective delivery of HIFU therapy
• Men with renal impairment with a GFR of < 35 ml/min (unable to tolerate gadolinium dynamic contrast enhanced MRI).

## Appendix B MRI protocol

### B.1 MRI conduct

#### B.1.1 General guidelines

a) A standard safety questionnaire should be completed.
b) For contrast enhancement: set up IV line in a vein in the antecubital fossa, connected to an automated injector with two syringes (contrast and saline flush).
c) 20 mg buscopan or 1 mg glucagon iv are *optional*, depending on local practice.
d) T2 sequences:
  Small field of view in 3 planes. The fields of view provided on the standard sequences are enough to cover most prostates. However, if the tips of seminal vesicles and the external sphincter cannot be included on the axial sequence, the number of slices (and with it the scan time) should be increased. In all cases the slice width should remain at 3 mm, with a 10% interslice gap.
e) Diffusion sequences (not included on early post HIFU scan):
  i) Multi-b with b values of 0,100,500 and 1000 s/mm^2^. 16 averages using a 3-trace technique. Standard Siemens algorithm for determination of ADC (currently includes b0 with monoexponential decay fitting, but this may be revised)
  ii) b1400 s/mm^2^ with 32 averages.
f) VIBE sequences:
  Dynamic contrast enhancement. Coverage should include the external sphincter and seminal vesicles as for the T2 axial sequences. If this cannot be ensured, the priority is to include the prostatic apex: the seminal vesicle tips are less important, as long as most of the vesicles are included. Contrast is 0.1 mmol/kg of low molecular weight gadolinium-based contrast: Magnevist or Dotarem (preferred in those with mild renal impairment), given at 3 ml/s. This should be followed by a flush of 20 ml normal saline. The infusion is started concurrently with the third dynamic acquisition. Acquisitions are continued for at least 5 min and 30 s after the start of the contrast infusion.

### B.2 5-Point Likert-type scoring system for mpMRI reporting

1 highly likely benign
2 likely benign
3 equivocal
4 likely malignant
5 highly likely malignant.
